# 

**Published:** 1871-10

**Authors:** 


### CONTENTS:

### ORIGINAL CONTRIBUTIONS.

The Unity or Duality of the Syphilitic Virus. By Edmund Andrews, M.D. 577
A Brief Sketch of a few Extraordinary Surgical Cases. By E. R. Willard, M.D., Wilmington, Ill.... 584
Typho-Malarial Fever, Complicated with Symptoms of Cerebro-Spinal Irritation. By N. S. Davis, M.D., Chicago ........ 588

### CLINICAL REPORTS.

Cases in the Medical Wards of Mercy Hospital. By Prof. N. S. Davis... 594
Cases in Mercy Hospital, Surgical Wards. By Prof Andrews ....... 601
Mercy Hospital — Opthalmological Ward. By Prof. Jones........... 602
Gynæcological Wards. By Prof. Byford........ 604
Biliary Calculi—Passage of a Large Number, and of Unusual Size. By N. S. Davis, M.D........ 605

### SELECTIONS.

Case of Muscular Atrophy. By V. Kersey, M.D........ 608
Thereaputical Actions and Uses of Turpentine........ 612
On the Element that Kills in Chloroform, and Other Allied Chemicals. By J. D. Brown, F.R.C.S. Eng.... 613
Varicella in San Francisco........ 619
Small-Pox in Philadelpia and Elsewhere........ 619
Climate of the Northwest and Nervous Diseases........ 620
Incision of the Elbow Joint to Relieve Anchylosis........ 621
British Medical Association—Papers Read, etc........ 621
Diagnosis of Phantom Abdominal Tumors........ 623
Acute Synovitis—Incision into the Joint........ 623
Excision of the Lateral Half of the Tongue........ 624
The Prussian Siege of Paris........ 624

### BOOK NOTICES.

A Memorial of Midwifery, etc. By Alfred Meadows, M.D........ 625
Handy-Book of the Treatment of Women's and Children's Diseases, etc. By Dr. Emil Dilluberger...... 626
A Treatise on Localized Electrization and its Application to Pathology and Therapeutics. By G. D. Duchenne.. 626
The Functions and Disorders of the Reproductive Organs in Childhood, etc. By William Acton, M.R.C.S.. 627
Practical Therapeutics, etc. By E. J. Warring, M.D. F.L.S........ 627
A Practical Treatise on Fractures and Dislocations. By F. H. Hamilton, A.M., M.D........ 628
Headaches. By H.G. Wright, M.D., etc 628

### EDITORIAL.

Explanation........ 629
Medical Colleges in Chicago........ 629
Effect of Chloral Hydrate on the Blood, 635
Publications—Notices of........ 638

